# Gastric ulcers in finishing pigs: the evaluation of selected non-dietary risk factors and impact on production performance

**DOI:** 10.1186/s40813-024-00362-0

**Published:** 2024-02-26

**Authors:** Piotr Cybulski, Aleksandra Woźniak, Magdalena Larska, Artur Jabłoński, Tomasz Stadejek

**Affiliations:** 1 Goodvalley Agro S.A, Dworcowa 25, 77-320 Przechlewo, Poland; 2 Department of Pathology and Veterinary Diagnostics, Institute of Veterinary Medicine, Warsaw University of Life Sciences - SGGW, Nowoursynowska 159C, 02-776 Warsaw, Poland; 3 Department of Virology, National Veterinary Research Institute, Partyzantów 57, 24-100 Puławy, Poland

**Keywords:** Pigs, Finishers, Gastric ulcers, Risk factors, Production parameters, Farm management

## Abstract

**Background:**

The complex aetiology of gastric lesions in pigs remains largely unknown and effective preventive measures and pharmaceutical treatment of the disease have not been developed yet. Regardless of the fact that the overwhelming majority of previous research works dealing with gastric ulceration in pigs focused on the role of the nutritional determinants, including chemical composition of feeds, cereal type, finely ground pelleted diets, and feed additives, conclusions presented therein remain highly ambiguous. Thus, the purpose of this study was to evaluate the impact of the disease on production performance, and investigate the influence of selected non-dietary risk factors on the prevalence of gastric alterations in finishing pigs reared under conditions of 11 modern farms located in Poland.

**Results:**

A total number of 26,043 finishing pigs was examined. 15,228 (58.47%) had gastric ulcers. Intact stomachs were detected in 6176 animals (23.71%). Parakeratosis and erosion were observed in 2551 (9.80%) and 2088 (8.02%), respectively. Among eight continuous variables two were found to be significantly associated with prevalence of the gastric ulcer: the growing number of animals in the herd, which was negatively correlated (*P* = 0.002; ρ = -0.37), and the growing average entry weight of animals transported to the finisher farm (*P* = 0.047; ρ = 0.24), which increased the risk of gastric ulcers prevalence. Among 12 nominal variables, problems with the quality of farm management (*P* = 0.041), and usage of straw as a bedding material (*P* = 0.002) were identified as determinants significantly associated with occurrence of the analysed health problem.

**Conclusions:**

Among 20 non-nutritional variables analysed in our study only few factors were found to be associated with the prevalence of the disease. The impact of broadly understood management issues on gastric health in finishing pigs deserves further research.

## Background

Species specific physiological properties of pig’s stomach underlie its susceptibility to the development of pathological alterations. The non-glandular interior part of the organ (*pars oesophagea s. pars nonglandularis tunicae mucosae*) surrounding the oesophageal opening is a relatively small area lined by stratified squamous epithelium. Negatively influenced by multiple damaging stimuli, its microanatomy favours the formation of alterations ranging from parakeratosis to erosions, and eventually, deep ulceration [1]. The complex aetiology of gastric lesions in pigs remains largely unknown and effective preventive measures and pharmaceutical treatment of the disease have not been developed yet [2–6].

Highly prevalent in all swine-rearing areas [7–12], the disease has exerted direct and tremendous impact on animal welfare and production economics [13, 14]. According to the available data, the mortality due to the disease accounts for 20–38% observed during an entire fattening period [14, 15]; whereas, in its second half, the disease is thought to be responsible for every second death [16]. Regardless of the focus on other production parameters, contradictory conclusions concerning the correlation between gastric ulcers and key performance indicators have been published to date [3, 11, 17–20].

Moreover, still relatively little is known about numerous environmental determinants, including the role of highly productive cross-bred finishers reared under conditions of intensive pig farming and various management issues. Even though poor farm management has been repeatedly reported by swine practitioners to have a tremendous impact on the development of gastric alterations in pigs, the reliable methods allowing objective assessment of the role of such events have not been described in the scientific literature. Besides, the vast majority of previous research works dealing with the broadly understood environmental on-farm stressors were issued a few decades ago [21]; therefore, the usefulness of such data might be severely limited, since livestock production technology and legal regulations on the animal welfare have undergone profound changes.

Following reasoning presented in scientific reports addressing human peptic ulcer disease [22, 23], the impact of *Helicobacter (H.)* spp. infection on swine was investigated so far. Although the presence of *H. suis* in pigs is very well documented, the conclusions regarding the specific role of the bacterium remain contradictory [24–26]. The research concerning the role of other microbes in the disease development, i.e. *Arcobacter* spp [27]., *Lactobacillus* spp., *Bacillus* spp [28]., *Fusobacterium (F.) gastrosuis* [29], or fungi [30], like *Candida* spp. are scarce and none of these infections is diagnosed and acknowledged as a highly specific risk factor triggering gastric ulceration in pigs. Nevertheless, the involvement of other pathogens causing common diseases of swine, including parasites, can be explained by the systemic effect of histamine, which stimulates H_2_ receptors and activates parietal cells to secrete hydrochloric acid [31]. Thus, it can be hypothesised, that any kind of treatment or prophylaxis against endemic diseases should reduce the prevalence of gastric ulcers in a swine herd. Regardless of the fact that the overwhelming majority of previous research works dealing with gastric ulceration in pigs focused on the role of the nutritional determinants, including chemical composition of feeds, cereal type, finely ground pelleted diets, and feed additives, conclusions presented therein remain highly ambiguous [32–37], what might be attributed to non-standardised research frameworks and considerable differences in the post-mortem classification of the gastric alterations. Thus, the purpose of this study was to evaluate the impact of the disease on production performance, and investigate the influence of selected non-dietary risk factors on the prevalence of gastric alterations in finishing pigs reared under conditions of 11 modern farms located in Poland.

## Results

A total number of 26,043 finishers was examined. 15,228 (58.47%) had gastric ulcers (grade 3) (Table 1). In the rest of animals, scores 1 (parakeratosis) and 2 (erosion) were observed in 2551 (9.80%) and 2088 (8.02%), respectively. Intact organs (grade 0) were detected in 6176 animals (23.71%).

**Table 1:** The prevalence of gastric lesions (n of observations = 68)

Score	Mean (%)	SD	Range in batches (%)
0 (intact organ)	23.43	14.80	0–63.62
1 (parakeratotic)	9.57	7.72	0–44.35
2 (erosion)	7.95	2.99	1.56–13.51
3 (ulcer)	59.24	15.49	18.55–89.77

The collected animal-level data was transformed to a herd-level database including 20 predictor variables allocated into five separated subcategories (i.e. environmental factors, production parameters, genetics, infections, prophylaxis and treatments) and the percentage of pigs with grade 3 as a response variable. The strength of the associations tested separately for all continuous (Table 2) and nominal (Table 3) explanatory variables revealed statistically significant correlation between 4 of them and gastric ulcers.

Among the eight continuous variables evaluated in our investigation, only two were found to be significantly (*P* < 0.05) associated with prevalence of the disease (Table 2). The growing herd size was recognised as a variable which significantly (*P* = 0.002) lowered the occurrence of gastric ulcers in the finisher farm. Similarly, the growing average entry weight of animals transported to the finisher farm was found to be significantly related to the greater occurrence of gastric ulcers in the herd (*P* = 0.047); however, the strength of linear relationship was defined as poor (ρ = 0.24).

**Table 2:** Descriptive statistics of the continuous variables and their association to the gastric ulcers prevalence in pig herds

Variable	Number of observations	Median	Min	Max	Spearman’s ρ	*P*
Environmental factors						
**Number of animals in the farm**	71	11,654	973	14,059	-0.37	**0.002**
Number of animals in the pen	71	25	25	550	-0.19	0.105
Production parameters						
Feeding days	70	87	61	118	-0.10	0.425
Mortality (%)	70	3.08	1.18	5.60	0.17	0.165
Average daily weight gain (g)	71	916	658	1116	0.01	0.960
Feed energy per kg (MJ)	71	2.85	2.40	3.39	0.18	0.123
**Average entry weight (kg)**	71	28.37	19.50	51.50	0.24	**0.047**
Live weight at slaughter (kg)	70	108.40	96.30	117.08	0.08	0.524

in bold - the variables significantly ( *P* < 0.05) associated with the prevalence of ulcers in finisher herds

Among 12 nominal variables, problems with the quality of farm management (*P* = 0.041) and usage of straw as a bedding material (*P* = 0.002) were identified as determinants significantly associated with high, and low occurrence of ulcers, respectively (Table 3). For the other 10 determinants allocated into four categories, i.e. production parameters (meteorological season, duration of the transportation to the slaughterhouse), genetics (PIC/DanBred), infections: porcine reproductive and respiratory syndrome virus (PRRSV), *Brachyspira (B.) hyodysenteriae*, *Mycoplasma (M.) hyponeumoniae*, and different *Salmonella* levels), prophylaxis and treatments (vaccination against intestinal lesions caused by *Lawsonia (L.) intracellularis*, deworming, and total antibiotic free production), statistically significant correlation was not demonstrated (*P* > 0.05).

**Table 3:** Descriptive statistics of the nominal predictor variables and their association to the gastric ulcers prevalence in pig herds

Variable (categories)	n_1_	n_n_	n_1_/n_n_^a^	*P*
Environmental factors				
**Management issues**	reported	not reported	6/65	**0.041**
**Flooring**	straw bedding	slatted floor	7/64	**0.002**
Duration of the transportation to the slaughterhouse	≤ 1 h	1-4 h	18/53	0.055
Meteorological season	winter	spring/summer/autumn	28/17/16/10	0.053
Genetics	PIC	DanBred	4/35	0.247
Infections				
Porcine reproductive and respiratory syndrome virus	no	yes	50/21	0.246
*Brachyspira hyodysenteriae*	no	yes	58/13	0.905
*Mycoplasma hyopneumoniae*	no	yes	4/67	0.319
*Salmonella* Index	1	2/3	33/15/18	0.128
Prophylaxis and treatments				
Vaccination against intestinal lesions caused by *Lawsonia intracellularis*	no	yes	50/12	0.473
Deworming	no	yes	70/1	0.130
Antibiotic free production	no	yes	70/1	0.107

^a^number of observations first category/number of observation following categories; in bold - the variables significantly (*P* < 0.05) associated with the prevalence of ulcers in finisher herds

## Discussion

The prevalence of the disease has been significantly increasing over the last 50 years; therefore, the consequences of gradual shift from extensive pig farming to the modern, intensive production systems are commonly thought to trigger the problem in question. Pigs on commercial farms (regardless of their capacity) are reared in artificially created populations and subpopulations, usually with an uniformed age structure, which is not found in natural conditions in any *Suidae* representatives. Despite commonly presented views on the social stress related to the number of animals occupying one pen and the frequency of gastric ulcers, our study has not confirmed it (*P* = 0.105); nevertheless, it should be borne in mind that the main limitation of the analysis is associated with the distribution in our data set in which the median and the minimum value are the same. Moreover, our result proving that the increasing farm size is related to the lower ulcer prevalence (*P* = 0.002; ρ = -0.37) directly contradicts the conclusions presented not only by previous authors [11, 38], but also the common and unjustified tendency to associate intensive swine farming with problems directly proportional to its size. Conflicting conclusions regarding the influence of increasing finisher farm size may be assigned to management conditions typical of large production systems. Indeed, secondary problems which stem from farm capacity, including the use of finely ground pelleted diets (regarded as the most ulcerogenic factor), and the purchase of animals from different suppliers (mixing pigs of different health status), tend to support the hypothesis being discussed.

It has been proven that rearing pigs on concrete slats increases the risk of the disease development almost four times compared to straw bedding [39]. Our results corroborate these findings. Constant access to deep straw bedding significantly reduced the occurrence of gastric ulcers (*P* = 0.002). Contemporary research have repeatedly indicated the gastroprotective effect of this material, both in terms of supplementation, and provision of deep bedding [40–44]. Attempts to explain the effect of straw on the lower occurrence of gastric alterations showed that supplementation at the level of 10, 500, or 1000 g (per animal per day, from 23 to 100 kg body weight) did not affect the frequency of parakeratosis and erosion. However, the daily administration of 500 or 1000 g reduced the ulcer prevalence [44]. Analysis of the effect of the material carried out in similar research framework revealed a curvilinear relationship between the supplementation and the lesions [45]. The negation of its gastroprotective effect was related mainly to the experimental frameworks providing insufficient supplementation of the material [46].

Available scientific studies have extensively described the role of fasting and temperature fluctuations on the induction of gastric lesions in pigs [2, 6, 21, 47, 48]. Our study proved that low quality of production management resulting in out-of-feed events, restrictions in access to water, late identification of diseased animals, or suboptimal thermal conditions was found to be significantly related to the frequency of gastric ulceration in pigs (*P* = 0.041). Even though the negative impact of management issues is well understood, the detection and determination of the importance of all the individual events violating animal welfare remain virtually impossible. It seems obvious, however, that the aforementioned problems noted in parallel (or in short intervals of time) may have acted synergistically.

Our result proving the lack of relationship between the disease and the length of transportation time to the slaughterhouse (*P* = 0.055) corroborates previously published data [11]. This parameter appears to be of importance in the disease pathogenesis only if considered as a vital element in a series of incidents forcibly preventing animals from sufficient feed consumption [49].

In the presented study the influence of seasonality on development of gastric ulcers was defined as statistically insignificant (*P* = 0.053) what can be ascribed to the systems providing perfectly stable conditions, irrespective of exposure to the most extreme occurrences of weather phenomena. Previous studies delineating the seasonality of the problem focused on increased mortality due to acute ulceration in two critical periods, summer and winter, linking hot weather with heat stress and reduced feed intake [50], or the higher frequency of porcine respiratory disease complex (PRDC) [13], respectively.

Regardless of the fact that advances in genetics and continuing improvement in production performance are thought to be highly conducive to ulcers, neither increased susceptibility to gastric lesions in highly productive pigs, nor specific ulcerogenic role of gradual growth in production parameters have been scientifically proved. In our study, no statistically significant relationship between the different swine genetics and the frequency of gastric ulceration in crossbred finishers was found (*P* = 0.247); however, one must bear in mind that the conclusion concerns only the comparison of two highly productive genetics (PIC and DanBred) reared under conditions of modern swine farming. There is no previous reports on such an observation in the peer-reviewed literature. Besides, the lack of control groups comprising non-industrial pig breeds kept under conditions of modern farming makes determination of the actual influence of genetic progress on the discussed problem virtually impossible. Moreover, targeting future research on such a juxtaposition is fundamentally impractical due to the sparse population of non-industrial breeds and their minor role in global pig farming.

Despite the lowest average daily gain (ADG) of the analysed batch (658 g) constituted approximately a half of the best result obtained in the study (1116 g), no significant correlation of the trait with the frequency of gastric ulcers was found (*P* = 0.960). The same observation applies to the number of feeding days (*P* = 0.425). The lack of association between gastric ulcers and growth rate in finishing pigs has been proved in previous studies [11, 19, 34, 51–53]. According to the cited authors, the high frequency of gastric lesions does not necessarily result in a significant growth retardation and economic performance reduction until increased mortality is observed. Nevertheless, there are studies presenting contradictory conclusions [17–3, 20, 54]. The negative impact of the disease on production parameters was determined in a study, where finishers with gastric ulcers reached from 900 to 1000 g, whereas pigs with no lesions, or developed parakeratosis, gained from 1000 to 1200 g a day [55]. Similar conclusions were presented by Ayles et al. [17] who demonstrated a negative correlation between increasing severity of gastric alterations and ADG, with the same observations applying to average daily feed intake. However, differences had occurred only in the initial stage of the disease and disappeared entirely at the time of development of moderate lesions (defined as a superficial erosion affecting more than 25% of non-glandular stomach, or a single deep erosion) and more advanced ones.

No statistically significant association between feed conversion rate (FCR) and the disease prevalence was found in our investigation (*P* = 0.123). Previous research works addressing FCR in context of gastric ulceration in finishers presented the same deduction [17, 20]. Similarly, mortality rate in finishers was not significantly associated with the occurrence of gastric ulcers (*P* = 0.165). The observation contradicts previously published data [15], and emphasises considerable differences in potential influence of the chronic and acute form of the disease in pig herds. Moreover, marked discrepancies may be partially attributed to the cumulative impact of several interfering factors not taken into consideration.

In our study, increasing average entry weight of the animals was found to be significantly associated with increased prevalence of the disease (*P* = 0.047; ρ = 0.24).Since the highest average entry weight of animals transported to the finisher farm was 51.5 kg, the result could have potentially been associated with inappropriate environmental conditions in the weaner farms. However, assessment of the clinical relevance of the management and housing conditions at the aforementioned locations was beyond the scope of our study. Having taken into consideration the lack of statistically significant association between the number of feeding days and the occurrence of gastric ulcers (*P* = 0.425), the observation might have been attributed to indetermined factors related to mixing, socialisation, and hierarchy formation in groups of relatively heavy animals; nevertheless, the identification of the *P-*value barely meeting the criterion for statistical significance indicates the need of additional systematic research.

The live weight at slaughter was statistically irrelevant (*P* = 0.524). The same inferences regarding the slaughter weight have been presented by other researchers, including the assessment of 70 kg animals in Australia (collectively referred to as porkers) [11], and an Italian piece of research demonstrating that rearing pigs up to 170 kg is not a risk factor contributing to the disease development per se [39].

In our study, no statistically significant correlation between the occurrence of *B. hyodysenteriae* infection and gastric ulcers in finishing pigs was found (*P* = 0.905). Considering the Latin name of swine dysentery (*gastrocolitis haemorrhagica necroticans suum*), clearly highlighting the negative influence of spirochaetes on the stomach, the role of *B. hyodysenteriae* in the development of gastric alterations should be taken into consideration. Nevertheless, the contemporary literature, contrary to the data presented in the historical veterinary handbooks, indicates the complete lack of molecular basis allowing previously mentioned reasoning, and describe the mechanism of *B. hyodysenteriae* pathogenesis only in the aspects of caecum and colon mucosa colonisation. Moreover, the scenario with the historical and/or local occurrence of highly specific *B. hyodysenteriae* strains is extremely unreliable. Hence, the gross pathological lesions observed by some authors in the organ (or just copied from prior works) should be considered a derivative of the chronic form of swine dysentery resulting in reduced feed intake and cachexia followed by ulceration.

Although PRRSV has been mentioned as one of the main factors inducing PRDC, peer-reviewed studies analysing its direct role in the pathogenesis of gastric ulcers are not available. Our investigation proved that PRRSV infection in fattening pigs (controlled by an administration of an attenuated vaccine) was not significantly correlated with the occurrence of gastric ulcers (*P* = 0.246). Similar observation apply to the lack of significant correlation between the occurrence of *M. hyopneumoniae* infection (controlled by a single dose of inactivated vaccine) and the disease (*P* = 0.319). Nevertheless, Italian researchers presented different conclusions addressing the impact of *M. hyopneumoniae* prophylaxis. According to their analysis, the vaccination against mycoplasmal pneumonia increases the risk of gastric ulcers, whereas, the prevention of other contagious diseases remains entirely irrelevant [39]. To avoid flawed reasoning, the authors regarded *M. hyopneumoniae* prophylaxis as a specific indicator of problematic herds, which, despite implementation of proper immunisation procedures, remain (for some unspecified reasons) not fully protected.

*Salmonella* spp. infections did not have a statistically significant relationship with gastric ulceration in our investigation (*P* = 0.128). Previous research on the correlation between the prevalence of *Salmonella* spp. and the occurrence of gastric lesions is lacking. *Salmonella* spp., similarly to other pathogens causing porcine enteropathies or just exacerbating their symptoms, is described therein only in a context of gastric ulcers differential diagnosis.

The vaccination against intestinal lesions caused by *Lawsonia intracellularis* infection was not significantly correlated with gastric ulcers (*P* = 0.473). The role of the immunisation in the aspect of the analysed problem has not been described to date. Similarly, there was no statistically significant correlation between the disease prevalence and the conventional and total antibiotic-free production (*P* = 0.107). This is the first report on this topic. While many authors indicated the potential bacterial aetiology of the disease [24, 26, 27], the use of different antimicrobials has not been described as having an impact on the prevalence of ulcers in swine herds [11, 56].

Routine deworming of fattening pigs remained without a statistically significant relationship with the disease prevalence (*P* = 0.130). According to available research works, the lack of deworming increases the risk of ulcers [39]. Since there is no active substance effectively acting against all stages of all pig parasites, the most likely reason for the divergent opinions is the non-standardised drug administration and various farm sanitation levels. Moreover, available scientific data demonstrate that only a few parasites can exert a direct influence on the stomach morphology, i.e. *Ascaris (A.) suum* [57], *Hyostrongylus rubidus* [58], *Ollulanus tricuspis* [59], *Ascarops* spp., *Physocephalus* spp., *Simondsia* spp [58]., *Gnathostoma* spp [58, 60].. Thus, the parasite life cycle causing multiorgan injuries followed by considerable increase in the level of histamine is much more likely than the local influence on the gastric tissue. This theory is supported by the successful ulcer induction by experimentally triggered migration of *A. suum* larvae, without the presence of adult forms in the stomach lumen [61].

## Conclusion

Despite the fact that ulceration of the non-glandular part of the stomach is highly prevalent and abattoir surveys provide valuable data on the incidence of gastric lesions in pigs, peer-reviewed research works delineating the role of non-nutritional factors in the disease development are scarce. Among twenty variables analysed in our study only few non-nutritional factors were found to be significantly associated with the prevalence of gastric ulcers. To summarise, the impact of broadly understood management on gastric health in finishing pigs reared under condition of modern farming, including objective assessment of the role of events potentially compromising their welfare, deserves further research.

## Materials and methods

This study was carried out on finishers slaughtered between January 2013 and February 2017 in a single abattoir located in Northern Poland. All the animals were reared in a three-phase production system using all-in all-out procedure and weekly batches. The PIC (PIC Group, Hendersonville, Tennessee, USA) and DanBred finishers (DanBred P/S, Ballerup, Denmark) were born after crossings ♀Camborough x ♂Line 19, and ♀(♂Landrace × ♀Yorkshire) × ♂Duroc, respectively. All the animals were reared in one of 11 farms belonging to a single production company and were offered unlimited access to dry steam conditioned pelleted feed (4 × 25 mm cylindrical pellet) supplied by the same feed mill. All the locations met the legal requirements of Council Directive 2008/120/EC of 18 December 2008 laying down minimum standards for the protection of pigs).

The farms enrolled in the investigation remained *Actinobacillus pleuropneumoniae*-negative, toxigenic *Pasteurella multocida*-negative, transmissible gastroenteritis virus (TGEV)-negative, and porcine epidemic diarrhoea virus (PEDV)-negative. All the sampled pigs were vaccinated against porcine circovirus 2 (PCV2)-associated diseases at the age of four weeks (Ingelvac CircoFLEX, Boehringer Ingelheim Vetmedica, Ingelheim am Rhein, Germany). In *Mycoplasma hyopneumoniae*-positive herds piglets were vaccinated at the same age using Ingelvac MycoFLEX (Boehringer Ingelheim Vetmedica, Ingelheim am Rhein, Germany).

In farms vaccinated against intestinal lesions caused by *Lawsonia intracellularis* infection weaners received Enterisol Ileitis (Boehringer Ingelheim Vetmedica, Ingelheim am Rhein, Germany) at the age of nine weeks. Animals transported to the PRRSV-positive farm were immunised using Porcilis PRRS (Intervet International B.V., Boxmeer, Netherlands) within a day after their arrival. The deworming treatment was completed using levamisole hydrochloride at the dose of 7.5 mg/kg body weight (Levamol 8%, Vetoquinol Biovet Sp. z o.o., Gorzów Wielkopolski, Poland). All the pigs reared in antibiotic-free production programme have never been treated with antibiotics (from birth to slaughter). To prove the traceability, pigs excluded from the program were immediately ear-tagged, moved to another group, transported, and slaughtered in a separated batch of animals.

The system evaluating the seroprevalence of *Salmonella* spp. was based on a monthly sampling of 7 meat juice samples collected from randomly selected batch of finishers originating from every farm. The herds were classified according to the proportion of seropositive samples collected during last 3 months into following *Salmonella* levels: level 1 (< 40%), level 2 (between 41% and 69%), or level 3 (> 70%). The analysis was performed in STANLAB Sp. z o.o (Nakło nad Notecią, Poland).

The presence of other pathogenic agents described in the study (*B. hyodysenteriae*, *M. hyopneumoniae*, and PRRSV) was defined before each slaughter investigation by a clinical examination of the herd carried out by the same swine veterinarian, followed by autopsies and routine laboratory tests. For *B. hyodysenteriae* detection (qPCR test), the diagnostic sample was a pooled faecal sample collected by a veterinarian from animals defecating abnormal stools. For *M. hyopneumoniae* (ELISA) and PRRSV (ELISA and qPCR) the diagnostic sample of choice was serum (15 samples collected from randomly selected finishers). All the analyses were performed in Weterynaryjne Laboratorium Diagnostyczne (Gietrzwałd, Poland).

In order to create an objective system allowing evaluation of farm management quality, four critical criteria were selected, i.e. restricted access to water (resulting from technical problems with nipple waterers), restricted access to feed (at least one empty feeder), late identification of animals requiring isolation and treatment, or inappropriate thermal conditions (defined as deviations from the environmental temperature curve causing evident behavioural problems). Identification of at least one of the listed faults by a veterinarian visiting the location allowed to qualify a batch of slaughtered animals as a batch exposed to the fundamental management mistakes. The veterinarian visits took place every week on a randomly selected working day.

Production reports were generated by the WinPig.Net management system (Agrosoft A/S, Hedensted, Denmark). The reliability of the reports was ensured by internal and external supervisors carrying out monthly audits involving stock inventory and test weighting of the animals. Slaughter weight was recorded from the abattoir internal system.

The finishers were transported to the slaughterhouse on trucksmeeting the requirements of Council Reregulation No 1/2005 of 22 December 2004 on the protection of animals during transport and related operations and amending Directives 64/432/EEC and 93/119/EC and Regulation No 1255/97. The feed was withdrawn for 12 h prior to the transportation. Depending on the farm location, transportation time varied from 15 min to 4 h. At the abattoir, the animals were stunned with carbon dioxide, suspended vertically, and bled out through the lower neck tissue.

Stomachs were opened along the greater curvature and examined 20 min post slaughter after expelling the digesta and rinsing with cold running water. Pathological alterations were graded by the same veterinarian using four-point scale outlined by Kopinski and McKenzie as follows [62]: grade 0– intact epithelium; 1– parakeratosis, 2– erosions, 3– developed ulcers. A total number of 26,043 organs was examined.

All the statistical analyses were performed using Statistica v.13.3 software (StatSoft Polska Sp. z o.o., Kraków, Poland). Spearman rank correlation and Kruskal-Wallis equality-of-populations rank test were used to identify relationships between the prevalence of gastric ulcers and continuous (number of animals in the farm, number of animals in the pen, feeding days, mortality, average daily gain, feed energy per kg, average entry weight, and average live wight at slaughter) and nominal variables (management issues, type of flooring, duration of the transportation to the slaughterhouse, meteorological season, genetics, occurrence of selected infections, and various prophylaxis and treatments), respectively. The *P*-value ≤ 0.05 was considered significant. The strength of linear relationship was interpreted according to Chan [63].

## Data Availability

The datasets used and analysed during the current study are available from the corresponding authors on reasonable request.
